# How does nursing-sensitive indicator feedback with nursing or interprofessional teams work and shape nursing performance improvement systems? A rapid realist review

**DOI:** 10.1186/s13643-022-02026-y

**Published:** 2022-08-24

**Authors:** Joachim Rapin, Joanie Pellet, Cédric Mabire, Sylvie Gendron, Carl-Ardy Dubois

**Affiliations:** 1 Faculty of Nursing, Université de Montréal, 2375 Chemin de la Côte-Sainte-Catherine, Montréal, Québec H3T 1A8 Canada; 2 Lausanne University Hospital, rue du Bugnon 21, CH - 1011 Lausanne, Switzerland; 3 Institute of Higher Education and Research in Healthcare – IUFRS, University of Lausanne, Biopôle 2 – Route de la Corniche 10, CH - 1010 Lausanne, Switzerland; 4 École de Santé Publique de L’Université de Montréal, 7101 Avenue du Parc, Montréal, Québec H3N 1X9 Canada

**Keywords:** Feedback, Nurses, Quality improvement, Quality of health care, Efficiency, Organizational, Outcome and process assessment, Health care, Review

## Abstract

**Background:**

Care quality varies between organizations and even units within an organization. Inadequate care can have harmful financial and social consequences, e.g. nosocomial infection, lengthened hospital stays or death. Experts recommend the implementation of nursing performance improvement systems to assess team performance and monitor patient outcomes as well as service efficiency. In practice, these systems provide nursing or interprofessional teams with nursing-sensitive indicator feedback. Feedback is essential since it commits teams to improve their practice, although it appears somewhat haphazard and, at times, overlooked. Research findings suggest that contextual dynamics, initial system performance and feedback modes interact in unknown ways. This rapid review aims to produce a theorization to explain what works in which contexts, and how feedback to nursing or interprofessional teams shape nursing performance improvement systems.

**Methods:**

Based on theory-driven realist methodology, with reference to an innovative combination of Actor-Network Theory and Critical Realist philosophy principles, this realist rapid review entailed an iterative procedure: 8766 documents in French and English, published between 2010 and 2018, were identified via 5 databases, and 23 were selected and analysed. Two expert panels (scientific and clinical) were consulted to improve the synthesis and systemic modelling of an original feedback theorization.

**Results:**

We identified three hypotheses, subdivided into twelve generative configurations to explain how feedback mobilizes nursing or interprofessional teams. Empirically founded and actionable, these propositions are supported by expert panels. Each configuration specifies contextualized mechanisms that explain feedback and team outcomes. Socially mediated mechanisms are particularly generative of action and agency.

**Conclusions:**

This rapid realist review provides an informative theoretical proposition to embrace the complexity of nursing-sensitive indicator feedback with nursing or interdisciplinary teams. Building on general explanations previously observed, this review provides insight into a deep explanation of feedback mechanisms.

**Systematic review registration:**

Prospero CRD42018110128.

**Supplementary Information:**

The online version contains supplementary material available at 10.1186/s13643-022-02026-y.

## Background

Feedback is an intervention designed to provide nursing or interprofessional teams with structured results about their performance [1]. For example, it is used by nursing performance improvement systems [NPIS]. NPIS measure a set of indicators that must be valid, reliable and sensitive to nursing care. It evaluates the performance of nursing services and aims to produce nursing or interprofessional improvement projects that generally target systemic change [2, 3]. Sharing results of performance measures with team members and decision makers can steer performance and improvement NPIS.

As a driven intervention, feedback involves the transmission and analysis of indicators, as well as the development of an action plan by clinical teams to improve their performance [4–6]. Feedback contributes to the engagement of nursing or interprofessional teams in practice improvement. However, feedback is also known to generate variable changes in service performance, and sometimes no changes at all, depending on the context. A systematic review of 140 randomized studies suggests that feedback improves health professionals’ (including nurses) compliance with desired clinical activities by 4.3% (adjusted weighted median difference in risk), with marked variability depending on the context [IIQ: 0.5 to 16%] [7]. These findings were echoed in a systematic review of seven randomized studies [8] which suggests that variability can be explained by (1) inconsistencies between feedback processes; (2) a range of contextual conditions, including service baseline performance [7]; or (3) the diversity of interactions between human and technical (i.e. sociotechnical) components [8]. These findings call feedback practices into question: social, technical and contextual interactions could explain the variability of the results.

Upon further examination, feedback appears to be conceptualized as a standardized intervention in a closed system, or, in other words, as a controlled or controllable technical intervention. For example, Ivers and Sales ([9], p. 1) define feedback as an intervention limited to who “can provide objective data regarding discrepancies between current practice and target performance, as well as comparisons of performance to other health professionals”. This textbook definition focuses on (1) the evaluation of professional practice at the expense of their mobilization and (2) the “objective” measurement of a standardized practice to be stringently applied regardless of context, as if professional practice were part of an established and standardized system. Yet, actions can be negotiated as, for example, in a conversation with a patient who does not want to undergo a recommended clinical route, or in the case of a specific health condition that would make the *desired clinical activity* useless for the health care team or a patient and his family. Current studies hardly mention the sociotechnical and contextual interactions that could help explain the variability observed impact, as a consequence of feedback interventions [10, 11].

In reality, feedback involves a number of processes, social interactions and techniques in different and unique contexts. These processes, interactions and contexts evolve and change over time and could explain the impact of feedback shared with nursing or interprofessional teams. For example, some teams may have transitional or lasting difficulty implementing planned feedback processes (e.g. cancellation, non-attendance due to sick leave) or mobilizing a concerted effort to improve their practices [12–15]. Analysis of indicators, interpretation of their meaning or the development of an action plan can generate controversies around emergent issues that require negotiation. Feedback is predicated on an unpredictable and complex interdependent social system. Actors have considerable leeway in their actions. At the same time, they are transformed and constrained by their spatiotemporal context and interactions [6, 16, 17]. These transformations and constraints can be identified in sociotechnical interactions. Technical components impact actors and transform their work over time. However, actors can also transform technical components. For example, data, including various clinical measures or observations, can be entered by clinicians, or during routine documentation of patients’ electronic records. These processes can be more or less user friendly: they can affect the reliability of results and even the engagement of nursing or interprofessional teams in the overall feedback activities. They can also be adapted to changes, for example, in how the data is collected or documented [18].

In short, feedback is a complex, interdependent and evolving social system of actors and various artefacts which, in reality, can be explained by sociotechnical and contextual interactions. However, the processes and interactions that are addressed in existing feedback theories and interventions are neither particularly dynamic nor contextualized, which is paradoxical considering the observed variability with respect to impact or changes generated by feedback interventions on nursing or interprofessional teams. The current state of research on feedback in NPISs limits our ability to understand (1) the occurrence and evolution of social interactions during feedback interventions with nursing or interprofessional teams, within their spatiotemporal context; (2) the evolution of sociotechnical interactions that combine human beings and specific technologies, within particular spatiotemporal contexts; and (3) the transformations generated by such a complex system of sociotechnical interactions. We postulate that a realist review will provide answers to these three limits.

The objective of this rapid review is to produce a theorization to explain what works in which contexts, and how feedback to nursing or interprofessional teams shape NPIS.

## Method

Realist reviews are based on realist philosophy principles [19]. Reality is envisioned as a set of complex systems, and reviews attempt to provide convincing explanations of observed dynamics. These explanations are transitive theories, since complex systems are in constant evolution. We therefore use the term “theorization,” since this is an open-ended process. We developed a theorization comprising demi-regularities of Context and Mechanism(s) =  > Outcome [C & M(s) =  > O] configurations [20]. Demi-regularities are demi-predictable patterns [21]: “demi” refers to interactions between actors and their context which generate variable results. The configuration C & M(s) =  > O suggests that context interacts with one or more mechanisms and produces outcomes that reflect transient states.

This rapid realist review complies with the publication standards for realist reviews [22]. The method is based on RAMESES recommendations [23]. Conducted over a 6-month period, as a preliminary phase to a realist study conducted in a Swiss teaching hospital, this proposal meets the criteria for a rapid realist review [24]. Our protocol was previously published [25]. The PRISMA 2020 checklist is provided in Additional file 1 [26].

### Design

The review was driven by six steps: (1) initial theory development, (2) search strategy, (3) selection and appraisal of documents, (4) data extraction, (5) analysis and synthesis and (6) presentation and dissemination of revised theory [23, 27]. This rapid realist review lays the groundwork for a theory on feedback in NPIS that subsequently informed a multiple case study.

#### Initial theory development

JR conducted an initial literature review to substantiate the background information and to identify potential logic models and middle range theories that could drive the review process [23, 28]. A logic model describes the study objects’ logic structure: the actors and processes leading to the outcomes. A middle range theory provides an initial explanation of how the intervention that will be studied during the realist review works.

This initial search relied on different databases (e.g. CINAHL, PubMed, Google Scholar) and an exploration through relevant articles (snowball strategy). On the one hand, Greenhalgh et al.’s logic model of “provider responses to performance data following feedback of ‘poor’ performance” [5] was selected, since it was the only one identified. It presents 12 interrelated processes such as: “ improve data collection, identify area of poor care, investigate cause or identifiy possible solutions” ([5], p. 22). This logic model initially allowed us to define and interrelate the different processes or actions involved in feedback.

Otherwise, considering the limitations of existing theories [5, 11], we combined [29] a theory with a philosophical approach, Actor-Network Theory [ANT] [30, 31] and Critical Realism [CR] [32], to address feedback as a complex sociotechnical system and to offer original theoretical foundations enhanced by their complementarity. This combination provides theoretical and heuristic parameters to address the three conceptual limits previously mentioned (including the lack of conception of social, technical and context interactions, and systems transformations). Some authors have suggested this combination [33, 34] without operationalizing it. Our operationalization is explained and justified in detail in our protocol [25].

Our approach to this review and general framework are based on CR and are expressed in the configuration C & M(s) =  > O. This configuration directs our search for explanatory underlying mechanisms: an in-depth (stratified) exploration of the complex system. ANT, on the other hand, is used to define the entities at work in the feedback system: intermediaries (e.g. documents, values, tools, resources or competencies that inscribe meaning into the system), actors (humans or non-humans that use or release intermediaries), mediators (actors who carry other actors) and networks that connect these different entities. ANT is therefore useful to inquire into feedback entities, changes in interelations, network reconfigurations and how actions are distributed between humans and non-humans [35]. Said otherwise, feedback, as a complex sociotechnical system, can be examined as made up of intermediaries, actors, networks, translations and mediators that are conceptualized with reference to ANT in order to operationalize C & M(s) =  > O configurations. ANT also provides insight into the connection and disconnection of entities that can be considered, in this complex sociotechnical system, as a result of translation operations. Specifically, feedback can be conceptualized as ANT translation operations: problematization (some actors are or become connected as they interact around emergent problems or issues that arise with feedback), interessement (some actors modify their identities, elaborate strategies, engage and connect or displace other actors to resolve problems or issues), enrolment (some actors then define and interconnect their roles to match their interests) and mobilization (a critical mass of actors becomes capable of coordinating their efforts to act together, actions are distributed among the actors of a network).

Key processes are inherent to these translation operations (e.g. controversy and convergence). These processes could explain the engagement of a critical mass of actors in the enhancement of their performance. “Controversies [tie] together and enmesh the techno-scientific and political contents that make up the issues facing actors” ([36], p. 176). Convergence is defined as:

the closure of controversies among actors that creates agreement among them and strengthens the network, stabilizing the [system]. Controversies are solved through translation by the addition of knowledge, other viewpoints and argumentative elements, as well as by the strengthening of existing connections and the enrolment of relevant new actors bringing new knowledge and resources necessary for action ([36], pp. 176–177).

For the purpose of this review, we defined these key processes as mechanism spaces to be investigated, in order to identify the feedback mechanisms at work [37]. For example, how did actors resolve a controversy raised by a nursing care-sensitive indicator (e.g. quantity of nursing staff supply) and initiate movement towards a shared understanding necessary to ensure commonly agreed upon adjustments in clinical practice?

**Fig. 1:**
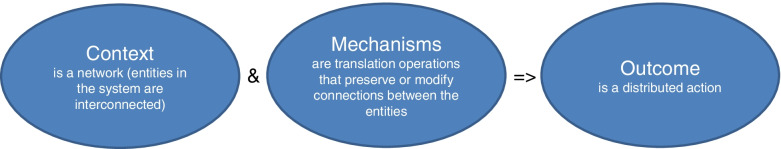
Model of the ANT-CR combination

Finally, given that C & M(s) =  > O configurations are traditionally presented as if (context) – then (outcome), with the specification of mechanisms. We developed a unique model of this configuration during this first phase of initial theory development, using ANT notions described above (Fig. 1).

This model presents the theoretical bases that oriented the following steps of this realist review: if entities are interconnected (context), then some of these entities can perform distributed actions (outcome), because their interactions may be the source of mechanisms, as translation operations, that preserve or modify connections between entities.

#### Search strategy

The search was directed at material (e.g. studies, review articles, concept papers, research reports and other relevant grey literature, websites or project initiation documents) published in English and French between 2010 and 2018, from the following databases: CINAHL, EMBASE, MEDLINE, Google Scholar and Web of Science (snowball strategy). The search strategy for CINAHL is published in the protocol [25] and the others in Additional file 2.

Since health systems, NPIS and feedback practices are constantly evolving, we selected a recent period i.e. last 8 years at the time this review was conducted. Although relevant information may be found in prior texts, resultant C & M(s) =  > O could reflect historical components that would have required more in-depth analysis. Such an analysis is hardly compatible with a rapid realist review. Otherwise, the databases were selected as these are considered classic sources for research on NPIS and feedback. Their number reflects the duration of this rapid review, which, in turn does not justify complementary or updated searches.

#### Selection and appraisal of documents

Selection of documents, literature search and data extraction (next section) were performed concurrently and iteratively [23]. The selection process proceeded as follows: (a) preliminary selection based on the title and summary of each document (by JR) and (b) final selection upon comprehensive reading of selected articles (by JR and JP). Inclusion criteria concern feedback intervention, population (at least nurses) and context (hospitals, outpatient services and residential facilities). For a document to be included, it had to provide (a) theoretical concepts or (b) empirical data. Documents about individualized feedback only were excluded. Reasons for exclusion were documented.

An appraisal form was developed by JR (Additional file 3) and tested on ten documents by two reviewers (JR and JP). Minor changes were made to enhance the form. Both reviewers read 40 articles, completed the form separately for each selected document and compared their results. Only one of the 40 documents required reexamination to reach compromise on appraisal. The form included the following information: article number and full reference, source database, country where the study was carried out, reasons for inclusion or exclusion and quality assessment. The two reviewers (JR and JP) assessed quality of each document based on two criteria: (a) relevance to the subject matter and (b) trustworthiness [23, 38]. Lastly, all included documents were examined by a pair of reviewers from the team (JP-CM, SG-JR or CAD-JR) to ensure reliability in their appraisal for inclusion. This examination presented no disagreement between the reviewers.

#### Data extraction

An extraction form was developed by JR (Additional file 4) and tested on ten documents by two reviewers (JR and JP). Minor changes were made to enhance the form. Two reviewers (JR and JP) extracted text excerpts from selected documents into a Microsoft Excel 2016® database that identified categories corresponding to ANT and CR concepts. Each section of the extraction form was systematically documented with information retrieved from each selected document. A pair of reviewers from the team (JP-CM, SG-JR or CAD-JR) examined the data extracted from all the documents to ensure greater reliability in data extraction. This examination presented no disagreement between the reviewers.

The data extracted from each document was also organized with reference to the logic model proposed by Greenhalgh et al. [5] (Additional file 5). It allowed us to identify feedback processes, interacting entities (e.g. intermediaries, actors, mediators, networks), documented outcomes (or results) and contexts.

#### Analysis and synthesis

Analysis started during the data extraction process. We identified controversies and convergences (e.g. mechanism spaces to be investigated) from each document, specifying (interpreting) the translation operations. An in-depth data analysis was performed through analytical questioning [39] and abductive reasoning to propose demi-regularities. Questioning the data included the following explorations: What are the controversies? What entities interact, connect and disconnect? What issues were identified by actors? How did actors close these controversies? What were the agreements or compromises? The construction and choice of demi-regularities was motivated by the search for the most plausible explanations, considering each controversy and convergence, as well as the outcomes and context. The iterative and systematic process of analytical questioning, combined with abductuve reasoning, resulted in the identification of plausible mechanisms. Thus, we formulated C & M(s) =  > O configurations that are coherent with our model of the ANT-CR combination (Fig. 1) and verified their consistency by returning to the documents.

To produce a synthesis, the resultant C & M(s) =  > O configurations were divided into three interrelated hypotheses. Each hypothesis groups several C & M(s) =  > O configurations, with the intent to differentiate the feedback system from the overall NPIS. The three hypotheses were then organized chronologically according to CR’s transformational model of social activity [40], which implies the pre-existence of a structure and its reproduction or transformation by individuals. The aim was to illustrate how a NPIS partially determines its feedback system, and how a feedback system reproduces or transforms the NPIS of which it is a part.

Lastly, the configurations, or explanatory chains, were compared and tested against the included documents. We relied on two criteria defined in our protocol: plausibility and consistency [38]. Writing this article also contributed to fine-tuning of our theorization. The process required that we go back to the original data to illustrate the configurations selected.

#### Expert consultations

Two expert panels were held in November 2018 [41]: one involving scientific experts from the Conseil consultatif sur la qualité et sécurité des soins of the Secrétariat international des infirmiers et des infirmières de l’espace francophone (SIDIIEF), and the other consisting of clinical experts from the Centre hospitalier universitaire vaudois (CHUV). The panels provided some specific additional information and ensured that our theorization made sense to the scientific and clinical panellists. Consistent with the principles of CR, this process supports the development of contextualized, plausible, coherent and understandable theorizations.

## Results

### Document flow diagram

**Fig. 2:**
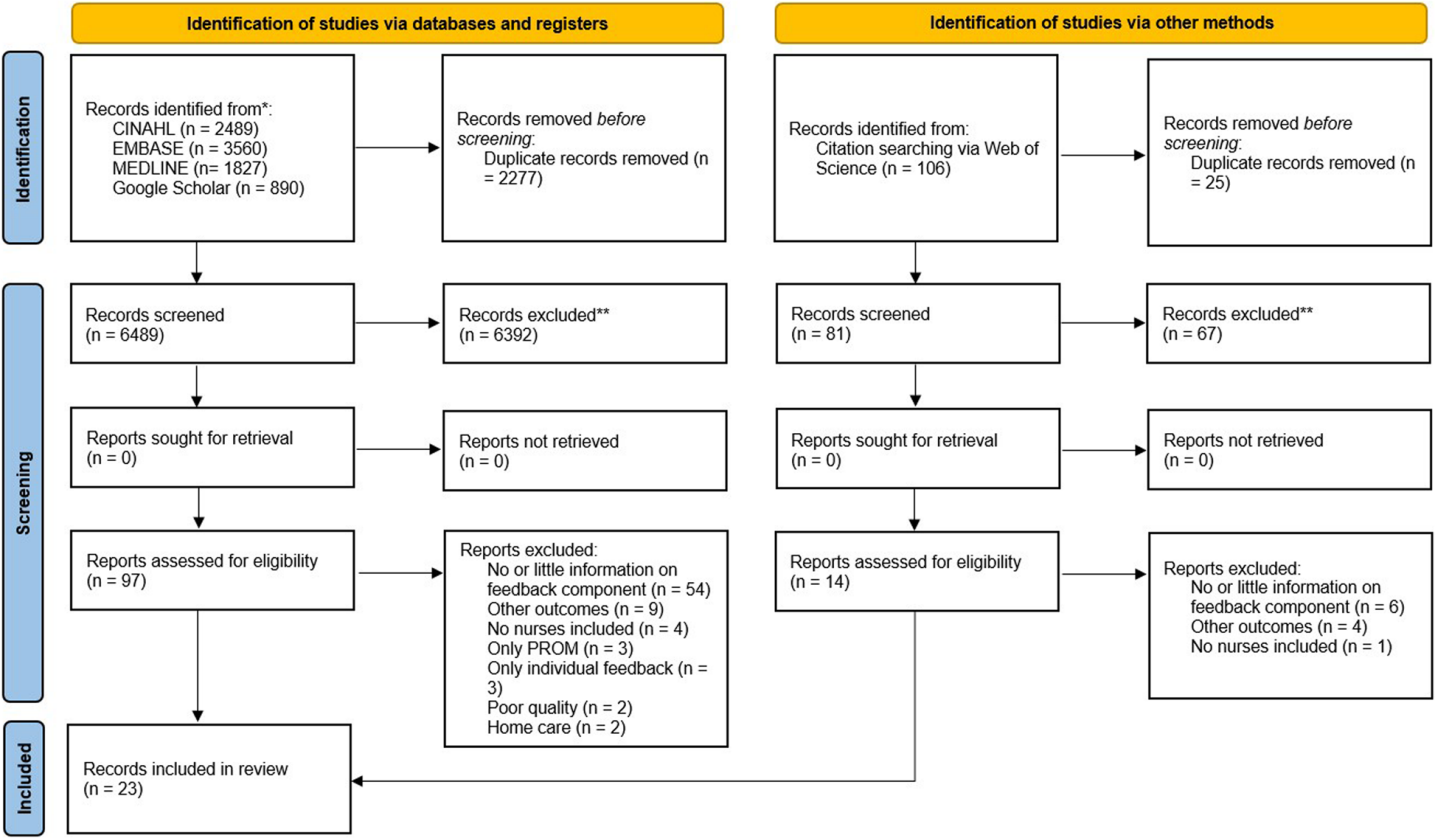
PRISMA 2020 flow diagram: NPIS feedback

From a pool of 8872 identified documents, 23 documents were included. The entire selection process is presented in the PRISMA 2020 flow diagram (Fig. 2) [26].

### Document characteristics

Of the twenty-three documents included in this review, six were published in Canada [10, 42–46], six in the Netherlands [13, 14, 47–50], four in the USA [12, 18, 51, 52], two in the UK [53, 54], one in Austria [15], one in New Zealand [55] and one in Australia [56]. Two are international articles [11, 57].

Eight studies are qualitative [e.g. semi-directed interviews, grounded theory] [11, 42–44, 46, 47, 50, 51], five are quantitative [e.g. randomized trials, cross-sectional surveys] [14, 15, 48, 49, 55], four are project reports [18, 52–54], three have mixed specifications [12, 13, 45] and three contain guidelines or recommendations [10, 56, 57].

With the exception of the last three documents and one study that identifies the different audit and feedback theories using semi-directed interviews [11], nineteen are related to the development or implementation of an NPIS [12–15, 18, 42–51, 53–55]. Thirteen articles concern hospitals [12, 13, 15, 18, 43, 46–48, 50–53, 55], four concern long-term care facilities [6, 18, 42, 45] and two pertain to outpatient clinics [14, 49].

### Main findings

We identified three hypotheses and twelve demi-regularities, which we specified in C & M(s) =  > O configurations. Hypothesis 1 includes 4 demi-regularities and C & M(s) =  > O configurations, Hypothesis 2 groups 7 demi-regularities and C & M(s) =  > O configurations and Hypothesis 3 presents one.

Hereafter, we describe each hypothesis and their demi-regularities briefly. For each demi-regularity, C & M(s) =  > O configurations are presented first. A descriptive presentation of the review and expert panel results that support each configuration then follows.

#### Hypothesis 1

An NPIS pre-exists feedback provided to nursing or interprofessional teams and partially determines to what extent these teams engage in feedback processes.

#### Demi-regularities

The NPIS contributes to the extent of the feedback intervention through (1.1) the appropriate choice of indicators and targets, (1.2) the appropriate choice of methods for transmitting indicator results, (1.3) understandable information and (1.4) the pre-existence of dense connections within NPIS networks.

##### Appropriate choice of indicators and targets

If the nursing or interprofessional teams participate in selecting indicators and targets [i.e. NPIS and teams are interrelated] (context), this will contribute to the engagement of clinical teams in feedback processes (outcome), because the intermediaries are aligned with team members’ identities, roles and practices (mechanism).

A number of documents underscore the importance, in practice, of indicators that are actionable or significant for professionals [10, 18, 44–46, 49, 50, 52, 54]. Some point out that indicators selected by nursing or interprofessional teams are particularly relevant [14, 18, 49, 52, 54, 55]. “The reports were explicitly designed to identify patients who needed specific actions and then were to be provided to individuals who, by virtue of the focus on actionable performance measures, would have the resources, knowledge, and authority to execute that action.” ([12], p. 879). Indicators and reports are intermediaries that give meaning to the NPIS. In this verbatim, these intermediaries connect with nursing or interprofessional teams through the consideration of their resources, knowledge, authority and practices. Indicators align with identities, roles and practices.

Targets can be chosen from different sources, for example, longitudinal results, benchmarking and scientific literature. Some NPIS or teams do not use targets, since there is not enough information on which to base their choice [10]. Nevertheless, many experts recommend the use of targets to reinforce desired practices [10, 56, 57] and to specify them in their NPIS [12, 13, 15, 18, 43, 44, 46, 47, 49–55].

Some targets may be critiqued for their lack of consideration of local contextual issues or patients [12, 13, 45]. Damschroder et al. [12] describe experiences of one-upmanship, each team trying to obtain better results than the others. Apparently, this situation prompted professionals to engage in practices without consideration for patients’ specific needs: they disconnected them. Moreover, these choices appear to have caused problems for some professionals, for example, when targets refer to standardized indicators: “We don’t always have a lot of ability to think outside the computer. It doesn’t allow for any real autonomy because if we don’t meet the measure, we’re going to get a letter” ([12], p. 880). When targets set standards for nursing or interprofessional teams, some team members may draw attention to their own capacity to generate significant measures and their autonomy, further prompting critical examination of their identities, roles and practices. Targets are thus intermediaries that give meaning to NPIS and feedback processes, with which the actors align themselves (or not).

##### Appropriate choice of methods for transmitting indicator results

If nursing or interprofessional teams choose methods for transmitting indicator results [i.e. NPIS and teams are interrelated] (context), this will contribute to team engagement in feedback (outcome), because transmissions and their realities are aligned (mechanism).

When results are not transmitted to teams on a continuous basis, or after long delays, or by inappropriate means, then transmission methods are not appropriate [13, 18, 45, 46, 51]. For example, as time passed, the results did not always seem to reflect current practice or outcomes. If feedback is transmitted too frequently, it can generate fatigue [10] and pressure to respond, and some teams can lose sight of its clinical significance [12]. It is recommended that a variety of transmission methods be used. However, there do not appear to be recommendations concerning the ideal frequency [10, 50, 57]. “Timeliness, in other words, was not just about the speed with which feedback was delivered but, more importantly, about a meaningful sense of temporality” ([46], p. 396). Transmission of indicator results (which are intermediaries) to nursing or interprofessional teams must be aligned with their expectations.

##### Understandable information

If results transmitted are understandable [i.e. NPIS and teams are interrelated] (context), this will contribute to nursing or interprofessional team engagement in feedback processes (outcome), because the information is aligned with their realities (mechanism).

Numerical results can be difficult for some professionals to understand, especially if they are presented in the form of advanced statistics, thus reducing actor engagement in the overall feedback process [52]. Level of training [18, 44, 48] and individual characteristics [11, 47–50] can influence capacity to understand transmitted information. However, this can be addressed in team discussions [49] or training sessions [52]. It is also recommended that results be presented in a clear and understandable manner that is adapted to team realities and preferences [10, 50, 52, 54, 56]. “Using this information, effects of audit and feedback can be improved by adapting the feedback format and contents to the preferences of stakeholders” ([50], p. 1276). Adapting the intermediaries to the actors requires that the NPIS connects and aligns with team members’ capacities and inclinations.

##### Pre-existence of dense connections within NPIS networks

If an NPIS is characterized by a formal, cross-disciplinary structure and dense connections between the networks [e.g. nursing or interdisciplinary teams, support teams, management] (context), this will contribute to engagement in feedback processes (outcome), because the values, identities and roles of most actors are aligned (mechanism).

Some NPISs appear to be institutionally organized and valued [43, 48, 52, 53]: they espouse a culture that promotes quality of care and patient safety. For instance:

The trust has a network of PdMs (Practice-development Matrons), who oversee the benchmark-scoring process in clinical areas. They work closely with any areas scoring red, supporting the ward sisters and charge nurses to plan for improvement and rescore the benchmarks within eight weeks. […] The trust’s patient-partnership group is integral to the process of developing each benchmark. […] In one directorate, the matron, the PdM and ward sisters and charge nurses developed specialty quality action teams. ([53], p. 23)

Such NPISs are integrated into every hierarchical level of the institution, in every unit and clinical network: connections between all these entities are dense. As multilayered systems, they include several intra- and extra-institutional networks, and the role of each actor and processes are defined [52, 53]. Although these NPISs may encounter strategic challenges or collaborative issues, they mobilize several networks (e.g. practice-development teams, nursing or interprofessional teams, patient groups) that appear to produce expected results [43, 52, 53] and that recognize the value and contribution of engaged actors to the NPIS, as well as to feedback processes [48, 52, 53].

In short, Hypothesis 1 presents a favourable context when an NPIS is connected to nursing or interprofessional teams in specific situations, thereby contributing to team engagement in feedback processes through mechanisms we referred to as the alignment of actor expectations, identities, roles and practices. The activation of such mechanisms strengthens the connections between the NPIS, the feedback system and nursing or interprofessional teams. In addition, it contributes to enrolment of clinical teams because it involves adherence (as in the selection of indicators or targets), which is a manifestation, among others, of consent towards NPIS.

#### Hypothesis 2

Feedback shared with nursing or interprofessional teams, through various operations that are activated (or not) concurrently (or not), potentially generates nursing performance improvements.

The (translation) operations include sequences of problematization, interessement, enrolment and mobilization of a critical mass of entities within nursing or interprofessional teams; and between these teams and other entities associated with the NPIS. Such sequences cannot be postulated per se. However, this realist review suggests tendencies for each of the operations.

Feedback generates problematization through (2.1) recognition of problems and (2.2) activation of values. Problematization results in a preliminary agreement on the nature of the problem to be resolved.

##### Recognition of problems

If a mediator presenting numerical results or indicators to a nursing or interprofessionnal team gives clinical meaning to the information, or identifies a discrepancy between the results presented and team expectations [i.e. mediator and teams are interrelated] (context), then teams can recognize the problem (outcome), because the mediator fosters a shift among actors (mechanism).

It has been observed that some actors, within a group, may identify a discrepancy between results presented and results desired as a problem [10, 41, 56, 57]. Compromise among all actors may not reached immediately when this occurs. Mediators can encourage a shift towards recognition of the problem among all the actors (if it really is a problem worthy of attention). Mediators can also prevent negative reactions to feedback [10, 11, 45, 52], such as indicators results not meeting their expectations. These shifts are generally accomplished by a manager or a respected member of the team [10, 11, 45, 52]. It may also be necessary for an actor, most often a local manager, to give clinical meaning to numerical results or indicators, for example, by describing an actual patient situation [41, 42, 47, 52]. “Story-telling can be a powerful way to bring data to life, and stories can make content more meaningful, actionable, and easy to grasp” ([52], p. 243). This “translation” by a mediator fosters a shift towards recognition of a problem among actors.

##### Activation of values

If problem identification can reduce team engagement or destroy team confidence and create a disconnect between actors (context), then a mediator can activate team values in order to avoid these consequences (outcome), because he or she can connect or strengthen connections among the values, the problem, the team and himself (mechanism).

In the case of contradictory results or indicators or in the face of an inconclusive action plan, some (team) actors may become demotivated or fear sanctions [41, 43, 56]. This is why NPIS mediators give frequent reminders of the value of feedback [non-punitive aspect, enhanced services and patient outcomes] [41, 52, 56]. It is also suggested that an effort be made to develop trust and respect in these conversations [10, 11, 56, 57]. “This person (mediator) should ideally be passionate about improving practice and have a good rapport with the critical care staff. Importantly, feedback needs to be timely, individualised and nonpunitive in order to be effective in improving performance.” ([56], p. 107). In this respect, some panellists also pointed out that the mediator responsible for implementing feedback in clinical teams should help support team analysis by systematically reminding all participants of the goals of feedback, whilst explaining the results, as needed [41]. This activation of values, such as respect or nonpunitive (as intermediaries), can prevent disconnection of actors from feedback processes and strengthen existing connections.

Feedback generates interessement through (2.3) introduction of additional information and (2.4) in-depth conversations and critical reflection. The result of interessement is agreement on possible solutions, which implies negotiations and interactions. During these processes, an intermediary is created to attempt to configure and solidify interactions between team members.

##### Introduction of additional information

If a mediator introduces supplementary data or facilitates the circulation of additional information about achievements and issues that have also arised in other teams (context), these intermediaries will foster negotiation and eventual agreement on possible solutions (outcome), because the connection of new intermediaries (by the mediator) will generate a shift within the nursing or interprofessional team (mechanism).

During feedback processes, some actors (mediators) decide to search for additional data in order to contextualize and further analyse performance measurement results or indicators for their nursing or interprofessional teams [12, 41, 43]. In these situations, all/most actors tend to agree on the identified problem [41]. This approach helps develop or strengthen agreement among actors, for example, with regard to the causes of the problem and, in addition, the actions that ought to be taken. In other words, these mediators introduce new intermediaries into the feedback processes. These new intermediaries generate negotiations, which can then lead to agreement and eventual repositioning towards envisioned (team) goals.

Institutions that support their clinical units through an external actor during feedback processes (e.g. a delegated manager or an institutional quality committee) foster: (1) in depth analysis of feedback results and improved tracking of planned action and (2) circulation of information about achievements and issues in other teams [52–55]. “[…], post review panel meetings were introduced to discuss results and support focused practice change and a continuous cycle of improvement activity. The programme’s executive group met with the charge nurse manager, divisional leader and selected unit staff” ([55], p. 2365). Thus, an external mediator can potentially introduce multiple intermediaries (in this example, a post review meetings), which generates further interactions, exchange of ideas, negotiation and compromise, which can eventually lead to an agreement—the latter being a condition for (potential) further interessement.

##### In-depth conversations and critical reflection

If nursing or interprofessional teams connected via in-depth conversations and critical reflection (context), then these conversations can lead to agreement concerning problems and solutions (outcome), because they create shared meaning, thereby adding further density to their connections.

Throughout feedback processes or team sessions, some nursing or interprofessional teams do not adequately complete their analysis or develop an action plan. In such cases, priority appears aimed at obtaining better results on performance measurements rather than establishing clinical relevance [12, 46]. This could, in part, explain why nursing or inteprofessional teams feel disconnected from feedback systems and, more so, marginalized and blamed [46].

Otherwise, some teams prefer or even recommend and organize forums to ensure in-depth conversations and critical reflection during the various feedback processes [10, 11, 42–46, 56]. “In this context, the conversations were critical and reflective in nature whereby nurses and managers would discuss why they were not achieving the outcomes they wanted for patient care” ([43], p. 1004). These conversations make it possible to identify difficulties or challenges, achieve expected results and come to a shared understanding. This approach strengthens connections.

Feedback generates enrolment through (2.5) agreement about the values inherent in the feedback processes and (2.6) compromise about the (best) plan to improve clinical practice. The result of enrolment is the expression of consent, which confirms alignment among actors. During these processes, an intermediary is created, and actors move to align with some other.

##### Agreement about the values inherent in the feedback system

If a mediator presents nursing or interprofessional teams with information that could render (potentially) opposing values significant values [i.e. intermediaries] (context), then these teams can align (create meaning) their different values (outcome), because the mediator, by connecting these intermediaries, encourages a shift in the nursing or interprofessional teams (mechanism).

Improving the quality of care is a value identified in all of the documents included in this review. However, this value competes with others, for example productivity. Competition between values can activate controversies [52]. Actors develop specific strategies to compose with a diversity of potentially opposing values, for example, communications, training or the creation of a concordance table [52, 53]. These strategies create intermediaries that mediators can connect to nursing or interprofessional teams. For instance:

Essence of Care provides a comprehensive review against best-practice standards and the Productive Ward initiative supports the review and redesign of the systems and processes involved. Links are also made from each benchmark tool to the Care Quality Commission’s (CQC, 2010) regulatory standards. This enables staff to build greater understanding of the connection between direct patient care and the national quality-assurance process ([53], p. 23).

In fact, such intermediaries can help teams reach a compromise on some values that give meaning to performance systems, external or internal to an institution. Mobilized by a mediator, they shift actors towards alignment between actors (e.g. management and clinical). In the following excerpt, feedback processes make it possible to understand quality and productivity issues, whilst also envisioning changes in practice that are aligned with nurses’ values with respect to their autonomy:

Data from health service dashboards establish a precise tracking system with credible benchmarks that drive action plans and strategies to contain costs while maintaining quality care. Moreover, access to these data by clinical nurses helps them understand trends in patient admissions and demographics, changes in their workload, and the need for more flexible self-scheduling procedures ([52], p. 238).

##### Compromise about the (best) plan to improve clinical practice

If a mediator and nursing or interprofessional team jointly develop a plan to improve clinical practice and outcomes (context), then there can be alignment on this plan (outcome 1) and implementation (outcome 2), because the network has reached a compromise on the (best) plan (mechanism).

Choosing goals, during or as a consequence of feedback sessions, involves political and strategic issues. These choices determine, at least in part, the scope of resultant clinical practice improvement plans. Nursing or interprofessional teams and local managers can experience controversy when local and institutional goals compete [12, 46, 52]. Compromise cannot always be reached, which can result in the disengagement of actors or the irrevocable termination of any planning process. The following excerpt suggests how some compromise on the goals to be achieved can be reached, whilst also acknowledging how external intermediaries influence their attainment: “These [goals] are selected from feedback given by clinical nurses, nurse leaders, physicians, and other health professionals; and regulatory requirements and national benchmarking programs influence goal attainment” ([52], p. 231). Otherwise, some managers who took part in the panels organized for this review pointed out that they systematically try to obtain their teams’ agreement and consent to determine priorities, training needs, required resources and goals to be attained. It is generally recommended that these aspects be included in a clinical practice improvement plan [41]. Thus, enrolment of nursing or interprofessional teams by their managers appears to foster adherence to improvement plans (intermediaries): the manager as mediator binds his team via a contract (i.e. improvement plans).

Feedback generates mobilization through (2.7) sustainable adjustment and alignment of goals and priorities. In turn, the result of mobilization is sustainable alignment and adjustment of the network of actors engaged, which is shaped by repeated compromises and distributed actions throughout the network and beyond.

##### Sustainable adjustment and alignment of goals and priorities

If nursing or interprofessional teams sustainably adjust and align their goals and priorities (context), then they can mobilize to improve their clinical practice (outcome), because they will have made repeated compromises concerning their goals and priorities (mechanism).

In an institutional setting, goals and priorities are systematically and periodically reviewed and adjusted as needed [52], based on performance measurements as well as ongoing connections between different internal and external networks. “Annually, priorities are reevaluated and new ones are identified using data-driven methodologies and input from nurses, physicians, and other health professionals who recognize the value of analyzing and tracking specific outcomes and performance measures.” ([52], p. 231). These sustainable adjustments and alignments can improve clinical practice and foster open and continuing communication channels with nursing or interprofessional teams [52]. Sustainability requires steady cooperation among various organizational networks that adjust and align their respective goals and priorities. As intermediaries, goals and priorities are adjusted and aligned between networks, the compromises arrived at represent sustainable, repeated distributed actions.

In summary, Hypothesis 2 represents a favourable context in which actors or a mediator introduce an intermediary into feedback processes. These intermediaries take different forms depending on the translation operations, for example, patient histories, the activation of values or additional data. Intermediaries give meaning to the feedback system. They also generate outcomes: problem recognition, negotiation and agreement, compromise and the sustainable adjustment and alignment of networks. These outcomes are generated through the shifting of actors or networks by a mediator.

#### Hypothesis 3

Engagement and mobilization of actors in feedback processes partially determine to what extent an NPIS changes (or not). Actor-determined action pre-exists change in an NPIS.

In particular, this review suggests that feedback helps change an NPIS through (3.1) the extension of intersystem mobilization.

##### Extension of intersystem mobilization

If nursing or interprofessional teams engaged in feedback processes achieve repeated success or clinical improvements (context), then their mobilization can be strengthened by an NPIS (outcome), because the teams’ actions densify connections within the feedback system, within the NPIS, and between the two systems (mechanism).

Repeated success of a feedback system, in particular in terms of higher performance results measured over time, leads to additional success, including enhanced engagement of actors in further clinical practice improvement [42, 43, 52].When such achievements are visible, they generate pride and motivation among actors involved in a feedback system [42, 43, 50–52, 55]. “Data become more relevant to practice when nurses realize that improvements in one quality indicator results in subsequent positive changes in others, and sometimes this impact leads to a ‘domino effect’” ([52], p. 240). In this way, actors develop and strengthen their connections within the feedback system and between the feedback system and the NPIS. Resource mobilization becomes tangible as quality indicator values increase or practices are improved, and translates into densified connections in the both feedback and NPIS networks.

Otherwise, NPIS in some institutions were upgraded as a consequence of problems identified in a feedback processes [13, 18, 54, 55]. For example, the frequency and method of transmitting or presenting performance indicators were modified after implementation of a feedback process, at the request of nursing or interprofessional teams. In turn, changing these intermediaries, in the NPIS, led to improvements in feedback processes. For example, teams became more engaged in feedback group work [18].

To sum up, Hypothesis 3 illustrates how the engagement of nursing or interprofessional teams in a feedback process generates changes in both the feedback system and in the overall NPIS. This engagement strengthens connections between human or non-human entities in a feedback system and in its NPIS, for example, by modifying intermediaries in response to meet clinical teams’ expectations.

**Fig. 3:**
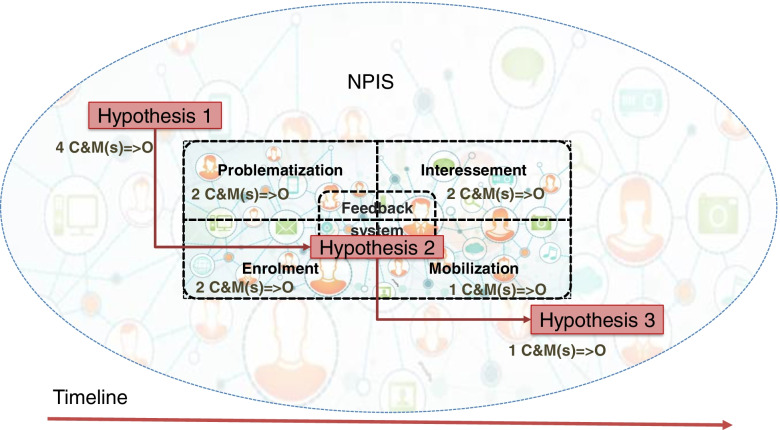
Model of the three hypotheses and twelve C & M(s) =  > O configurations

Figure 3 offers a synthesis of our realist review and illustrates an NPIS and its feedback system on a time continuum. It presents the three hypotheses, each with its C & M(s) =  > O configurations. The four ANT translation operations are portrayed in the feedback system. The interprofessional sociotechnical networks are illustrated in an overlay.

The hypotheses, demi-regularities and C & M(s) =  > O configurations are summarized in Table 1.

**Table 1:** Hypotheses, demi-regularities and C & M(s) =  > O

Hypotheses	Demi-regularities	C & M(s) = > O configurations synthesis
Hypothesis 1: An NPIS pre-exists feedback provided to nursing or interprofessional teams and partially determines to what extent these teams engage in the feedback process	1 The NPIS contributes to the extent of the feedback through 1.1 Appropriate choice of indicators and targets 1.2 Appropriate choice of a method for transmitting indicator results 1.3 Understandable information 1.4 Pre-existence of dense connections within NPIS networks	Context: NPIS is connected to the nurse or interprofessional teams in specific situations Outcomes: It contributes to their engagement and adherence in the feedback process Mechanisms: The NPIS is aligned with the actors’ expectations, identities, roles and practices
Hypothesis 2: Feedback shared with nursing or interprofessional teams, through various operations that are activated (or not) concurrently (or not), potentially generates nursing performance improvements	2 Feedback generates ***problematization*** through 2.1 Recognition of problems 2.2 Activation of values Feedback generates ***interessement*** through 2.3 Introduction of additional information 2.4 In-depth conversations and critical reflection Feedback generates ***enrolment*** through 2.5 Agreement about the values inherent in the feedback processes 2.6 Compromise about the (best) plan to improve clinical practice Feedback generates ***mobilization*** through 2.7 Sustainable adjustment and alignment of goals and priorities	Context: Actors or mediators introduce intermediaries (e.g. value, new information, space for critical reflection) that give meaning to the system Outcomes: There is recognition of problems, negotiation and convergence, compromise and the sustainable adjustment and alignment of the networks Mechanisms: Actors or networks are shift by a mediator
Hypothesis 3: Engagement and mobilization of actors in the feedback processes partially determine to what extent the NPIS changes (or not)	3 Feedback contributes to the transformation of the NPIS through an extension of intersystem mobilization	Context: Actors engaged in the feedback process achieve repeated success or improvements Outcomes: Their mobilization can be strengthened by the NPIS Mechanism: Actors create denser connections within and between the feedback system and the NPIS

## Discussion

### Summary of findings

This rapid realist review conceptualizes how social and sociotechnical interactions develop and evolve, in context, through NPIS feedback processes engaged with nursing or interprofessional teams. Moreover, this review provides insight into the co-evolution and change dynamics of these imbricated systems, NPIS and feedback, both involving human and technical entities.

Beyond technical considerations inherent to feedback processes, Hypothesis 1 suggests that human actors’ expectations, identities, roles and practices partially determine to what extent they actually engage in a feedback process. Hypothesis 2 uncovers how human interactions evolve through problematization, negotiation and compromise. Hypothesis 3 addresses the mobilization of actors, revealing the development of their connections, as well as the recursive dynamics that potentially extend these connections within a feedback system and between a feedback system and its NPIS. The three hypotheses point to ongoing repositioning of actors in a feedback system, through dynamic re-connections or disconnections. These movements and rearrangements are generated by mediators, who are usually managers or trusted and respected professionals.

In sociotechnical terms, the three hypotheses highlight the role of intermediaries and their interactions with actors. For example, reports and dashboards are hybrid intermediaries that introduce performance measurement results, clinical targets and system values. These intermediaries give meaning to feedback systems and NPIS and participate in the repositioning of actors, the strengthening of connections or their disconnection or rearrangements, depending on the context.

Each hypothesis illustrates transformation that occurs in NPIS and feedback systems. The first hypothesis describes how NPIS changes through sociotechnical interactions and how it can transform feedback processes. The second hypothesis illustrates how feedback changes through translation operations. The third hypothesis illustrates how feedback transforms NPIS through systemic changes. The resultant theorization (Fig. 3) illustrates how feedback systems and NPIS interact, and co-evolve as they are recursively transformed. The three hypotheses are thus interrelated, not in a linear fashion, but through systemic, iterative and recursive interactions: system transformations are generated by mechanisms and their (spatio-temporal) context.

Given the above hypotheses, it appears that the variability that is repeatedly documented with respect to the results of feedback interventions with nursing or interprofessionnal teams is not a methodological problem. The variability is inherent to these sociotechnical systems. It is in the nature of these systems that develop, evolve and transform over time. How then do we ensure that feedback generates changes that ultimately improve care? This rapid literature review offers a conceptual map to support further research, with an aim to enhance feedback processes with nursing or interprofessional teams.

### Comparison with existing literature

We did not find a comparable theorization to the one we propose in this review. To our knowledge, one retrieved document proposes seven explanatory feedback mechanisms to consider: (1) a complex feedback cycle process, (2) relative advantage, (3) resource match, (4) compatibility, (5) credibility, (6) social influence and (7) actionability [6]. These mechanisms are not related to specific contexts. The critical realist C & M(s) =  > O configuration, combined with ANT concepts, has proven useful in this review to engage in an in-depth explanation of feedback processes. In addition, the proposed synthesis offers insight into specific mechanisms that require further exploration to enhance feedback systems with nursing or interprofessional teams.

Recent literature reviews and theoretical frameworks not included in this review are, nevertheless, consistent with some mechanisms included in our results: (a) prior participation of nursing or interprofessional teams in the development of feedback processes (e.g. choice of data presentation formats) [4, 58–61]; (b) structure, frequency, content and goals of feedback processes [60, 61]; (c) development of trust and respect between actors engaged in feedback [4, 60]; and (d) contextualizing performance measurement results, reflecting on the meaning of indicators, sharing experiences [4, 59]. Other recommendations include the need to address barriers and facilitators to change and ensuring the development of clear action plans [60]. However, the latter do not make explicit the engagement of clinicians or managers to whom feedback is addressed.

For researchers situated in a realist approach, a pre-existing programme theory or middle range theory is an expected scientific and methodological requirement [62]. To create an initial theory for our proposed research, two theories [29] and data sources (e.g. focus group and literature review) [24] were combined. The CR-ANT combination used for this review appears original at this time, since feedback is conceptualized as a complex system, rather than as a pre-determined programme or intervention, and it provides a particular orientation to the conception of C & M(s) =  > O configurations. This review proposes an alternative and innovative conceptualization of feedback processes that can be used as a methodological tool to enhance further development of feedback practices with nursing or interprofessional teams.

Further efforts are required, however, to refine our understanding and conceptualization of “context” and “mechanisms”, given their polysemic potential. For instance, two types of “context” have been proposed for realist researchers, based on (a) observable characteristics (space, place, person, thing) or (b) relational and dynamic characteristics [63]. In this review, our definition of context refers to the second type. With respect to the idea of “mechanisms”, there are several definitions, and ongoing conceptual and methodological debates [64]. Two propositions formulated by RAMESES are coherent with our approach: “a) an explanatory account of how and why programmes give rise to outcomes, and (b) hidden, but still real, shaped by and interconnected with context” ([65], p. 2). A third characteristic of mechanisms offered by RAMESES, “the interaction between program resources and the ways that participants interpret and respond (or not) to them”, is shaped by programme-based approach. It remains that the definition of essential terms that refer to resources, interpretation and responses remains an open question. In this review, our definition of mechanisms is clearly shaped by ANT and could contribute to current discussions.

### Strengths, limitations and future research directions

The main strength of this review is that it offers in-depth understanding, through 12 contextualized mechanisms, of the variable outcomes observed when feedback on performance indicators is shared with nursing or interprofessional teams. This review adds to current research findings that tend to focus on technical aspects of feedback processes with clinical teams, and opens an area of enquiry at the junction of humanities, social and health sciences in an attempt to develop actionable knowledge. The panels of experts that participated in this review were instrumental in bridging theoretical ideas with practice by identifying the most important clinical issues, as well as the most relevant and actionable ideas to enhance feedback processes with clinical teams and foster their engagement in NPIS.

Furthermore, our CR-ANT device made it possible to conceptualize the complexity of feedback processes. As a methodological tool drawing attention to controversy and agreement related mechanisms, this device facilitated the identification and theorizing of key pathways that could explain both feedback system and NPIS transformation, as well as their sociotechnical interactions over time. Our methodological tool is consistent with realist ontology. According to Archer [66], ontology plays a regulatory role on methodology. In this review, our conception of social reality influenced our theorization of techniques used in feedback systems. In the end, the results of this review provide a theory of feedback, as it evolves (or not) in reality, although there is no claim to conflate our theory with reality.

In terms of limitations, it is worth noting that some of the documents retrieved for this review barely addressed contextual specificities or offered minimal description of the feedback processes under study. Some pertinent information was found in other sources, although it is possible that tacit or experiential knowledge may have biased some interpretations, despite our internal validation procedures described above. In addition, most of the documents do not attend to chronological description. For example, the articulation of strategies used to connect entities, in/from their respective contexts, was poorly described, which limited our ability to engage in a deeper exploration of the mechanisms potentially at work. Finally, individualized feedback processes, deployed on a one-to-one basis, as well as feedback interventions implemented in other organizations that do not include nurses, were excluded from this rapid realist review. It remains to be seen if a further realist review including these features would prove relevant.

With respect to future research, it is striking that artificial intelligence apparatus were not identified in this review. Current developments could modify our theorization and open novel research avenues with respect to sociotechnical system interactions.

### Recommendations

For clinical practice or education, we recommend using an explanatory theory of feedback with nursing or interprofessional teams as a conceptual model to enhance reflexive capacity with engaged stakeholders. Although standardized feedback interventions are perceived as a sign of quality, this review suggests that a systemic model of contextualized mechanisms may enhance deeper understanding of the reality of feedback as *it is* and that will continue to produce variable outcomes.

For research, taking into account the above-mentioned limitations, in-depth studies of real feedback systems could significantly enrich this theorization. We recommend that researchers describe interventions in detail, in their publications, in order to facilitate readers’ understanding of what works, for whom, how and in what context. The 54 documents that were excluded from this review as a consequence of anecdotal description, could conceal unidentified mechanisms that are at work. Lastly, we also recommend using coherent theoretical and methodological tools to address the complexity of health contexts.

## Conclusion

This rapid realist review provides a transitive and sociotechnical explanation of feedback processes engaged with nursing or interprofessional teams that conceptualizes spatiotemporal context, as well as technical and human interactions. It provides an innovative explanation of complex sociotechnical feedback systems and their variable outcomes.

## Supplementary Information

**Additional file 1.** PRISMA Checklist.

**Additional file 2.** Search strategy in MEDLINE; EMBASE & Google Scholar.

**Additional file 3.** Appraisal form.

**Additional file 4.** Extraction form.

**Additional file 5.** Greenhalgh et al.’s logic model.

## Data Availability

The datasets used and analysed during the current study are available from the corresponding author on reasonable request.

## Abbreviations

ANT: Actor-Network Theory
C & M(s) = > O: Context and Mechanism(s) = > Outcome
CHUV: Centre hospitalier universitaire vaudois
CR: Critical realism
NPIS: Nursing performance improvement system
SIDIIEF: Secrétariat international des infirmiers et des infirmières de l’espace francophone
