# Supplementary material for: How does nursing-sensitive indicator feedback with nursing or interprofessional teams work and shape nursing performance improvement systems? A rapid realist review
Source: Syst Rev. 2022 Aug 24;11:177. doi: 10.1186/s13643-022-02026-y (PMC9404638; doi:10.1186/s13643-022-02026-y)
Supplement: Supplementary file 2 — Additional file 2. Search strategy in MEDLINE; EMBASE & Google Scholar. [file 13643_2022_2026_MOESM2_ESM.docx]

**Additional file 2**

*Search strategy in MEDLINE (searched 30.08.2018)*

| [# ▲](http://ovidsp.tx.ovid.com/sp-3.31.1b/ovidweb.cgi?&S=AJLPFPHBFIDDJHOLNCEKPDJCNPOLAA00&Sort+Sets=descending) | **Searches** |
| --- | --- |
| 1 | "quality of health care"/ or advance directive adherence/ or guideline adherence/ or exp "Outcome and Process Assessment (Health Care)"/ or treatment outcome/ or treatment failure/ or "process assessment (health care)"/ or peer review, health care/ or "professional review organizations"/ or program evaluation/ or benchmarking/ or quality assurance, health care/ or clinical audit/ or nursing audit/ or total quality management/ or exp quality improvement/ or exp quality indicators, health care/ or "utilization review"/ or accreditation/ or "facility regulation and control"/ or time out, healthcare/ or "health care quality, access, and evaluation"/ or health care evaluation mechanisms/ |
| 2 | Clinical Governance/ |
| 3 | Efficiency, Organizational/ |
| 4 | Organizational Innovation/ |
| 5 | "Joint Commission on Accreditation of Healthcare Organizations"/ |
| 6 | "peer review"/ or peer review, health care/ |
| 7 | "Root Cause Analysis"/ |
| 8 | Social Responsibility/ |
| 9 | 1 or 2 or 3 or 4 or 5 or 6 or 7 or 8 |
| 10 | LICENSED PRACTICAL NURSES/ or exp NURSES/ or NURSE'S ROLE/ |
| 11 | exp NURSING CARE/ or exp NURSING PROCESS/ or exp NURSING/ |
| 12 | exp Nursing Staff/ |
| 13 | 10 or 11 or 12 |
| 14 | FEEDBACK, PSYCHOLOGICAL/ or FEEDBACK/ or FORMATIVE FEEDBACK/ |
| 15 | 9 and 13 and 14 |
| 16 | (((Perform* or quality) adj2 (measur* or manag* or improv* or indicator* or program* or system* or assess* or evaluat* or care or health* or organi?at*)) or (Outcome? adj2 (assess* or evaluat* or nurs* or treatment or care or health*)) or (clinical* adj2 (indicator* or governance or effective* or efficien*)) or (organi?at* adj3 (effective* or efficien* or change or innovation)) or (guideline* adj2 adher*) or "joint commission" or " JCAHO" or "Health Plan Employer Data and Information Set" or "HEDIS" or audit* or "peer review*" or ("process assessment" adj2 health*) or "Root Cause Analysis" or "Variance Analysis" or (program* adj2 evaluat*) or (organi?ation* adj2 accountabilit*) or benchmarking).ab,hw,kf,kw,sh,ti. |
| 17 | (Feedback* or "feed-back*").ab,hw,kf,kw,sh,ti. |
| 18 | "nurs*".af. |
| 19 | 16 and 17 and 18 |
| 20 | 15 or 19 |

*Search strategy in EMBASE (searched 30.08.2018)*

| [# ▲](http://ovidsp.tx.ovid.com/sp-3.31.1b/ovidweb.cgi?&S=AJLPFPHBFIDDJHOLNCEKPDJCNPOLAA00&Sort+Sets=descending) | **Searches** |
| --- | --- |
| 1 | health care quality/ or benchmarking/ or clinical effectiveness/ or clinical indicator/ or nursing outcome/ or "quality of nursing care"/ or "root cause analysis"/ |
| 2 | exp performance measurement system/ or exp program evaluation/ or "utilization review"/ |
| 3 | treatment outcome/ or clinical outcome/ or outcome assessment/ or patient-reported outcome/ or treatment failure/ |
| 4 | total quality management/ |
| 5 | quality control/ or clinical audit/ or nursing audit/ or quality circle/ |
| 6 | organizational development/ or organizational efficiency/ |
| 7 | protocol compliance/ |
| 8 | accreditation/ |
| 9 | "peer review"/ or "peer review organization"/ or "professional standards review organization"/ |
| 10 | 1 or 2 or 3 or 4 or 5 or 6 or 7 or 8 or 9 |
| 11 | negative feedback/ or positive feedback/ or psychological feedback/ |
| 12 | exp nursing/ |
| 13 | exp nurse/ |
| 14 | exp nursing care/ |
| 15 | 12 or 13 or 14 |
| 16 | 10 and 11 and 15 |
| 17 | (((Perform* or quality) adj2 (measur* or manag* or improv* or indicator* or program* or system* or assess* or evaluat* or care or health* or organi?at*)) or (Outcome? adj2 (assess* or evaluat* or nurs* or treatment or care or health*)) or (clinical* adj2 (indicator* or governance or effective* or efficien*)) or (organi?at* adj3 (effective* or efficien* or change or innovation)) or (guideline* adj2 adher*) or "joint commission" or " JCAHO" or "Health Plan Employer Data and Information Set" or "HEDIS" or audit* or "peer review*" or ("process assessment" adj2 health*) or "Root Cause Analysis" or "Variance Analysis" or (program* adj2 evaluat*) or (organi?ation* adj2 accountabilit*) or benchmarking).ab,hw,kw,sh,ti. |
| 18 | (Feedback* or "feed-back*").ab,hw,kw,sh,ti. |
| 19 | "nurs*".af. |
| 20 | 17 and 18 and 19 |
| 21 | 16 or 20 |

*Search strategy in Google Scholar (searched 24.07.2018, the first 10 pages)*

***nurs**** feedback performance measurement systems

nurs* feedback performance management

nurs* feedback performance improvement

nurs* feedback program performance

nurs* feedback quality improvement

nurs* feedback quality assurance

nurs* feedback quality improvement programs

nurs* feedback quality improvement systems

nurs* feedback quality measurement

nurs* feedback quality indicators

nurs* feedback quality assessment

nurs* feedback quality of Health Care

nurs* feedback quality Management Organizational

nurs* feedback outcome assessment

nurs* feedback outcome evaluation

nurs* feedback Nursing outcomes

nurs* feedback Treatment outcomes

nurs* feedback Healthcare outcomes

nurs* feedback Care outcomes

nurs* feedback Clinical indicators

nurs* feedback Clinical Governance

nurs* feedback Clinical Effectiveness

nurs* feedback Organizational Efficiency

nurs* feedback Organizational change

nurs* feedback Organizational innovation

nurs* feedback Effective organisational strategies

nurs* feedback Guideline Adherence

nurs* feedback Joint Commission Core Measures

nurs* feedback Joint Commission

nurs* feedback Joint commission on accredidation of health care organizations

nurs* feedback Health Plan Employer Data and Information Set

nurs* feedback Nursing Audit

nurs* feedback Peer Review

nurs* feedback Peer Review organizations

nurs* feedback Process Assessment Health Care

nurs* feedback Root Cause Analysis

nurs* feedback Variance Analysis

nurs* feedback Program Evaluation

nurs* feedback Organizations accountability

nurs* feedback Benchmarking
