# Supplementary material for: How does nursing-sensitive indicator feedback with nursing or interprofessional teams work and shape nursing performance improvement systems? A rapid realist review
Source: Syst Rev. 2022 Aug 24;11:177. doi: 10.1186/s13643-022-02026-y (PMC9404638; doi:10.1186/s13643-022-02026-y)
Supplement: Supplementary file 3 — Additional file 3. Appraisal form. [file 13643_2022_2026_MOESM3_ESM.docx]

**Additional file 3**

*Appraisal form*

| Article number |  |
| --- | --- |
| Complete reference |  |
| Target country |  |
| Article source |  |
| Inclusion criteria *General* | A) Contributes to program theory |
|  | B) Helps test the elements of program theory |
|  | C) Off-topic article (exclusion) |
| Inclusion criteria *Health context addressed in the article* | a) Hospital |
|  | b) Outpatient clinics (CLSC, private medical clinic, etc.) |
|  | c) Long-term care facilities |
|  | d) Other (if only other: exclusion) Specify: |
| Inclusion criteria *Dubois et al. (2013) indicators* | Article addresses Dubois et al. (2013) indicators |
|  | d) Other indicators (if only other: exclusion) |
| Exclusion criteria | Article addresses only PROM |
|  | Article addresses only individual feedback |
| DECISION | Included = 1; Excluded = 0 |
| Study type |  |
| Study design |  |
|  |  |
| Rigorous criteria | Transparency: explanation of research process (detailed and comprehensive) |
|  | Reliability of data (Wong, 2018) |
|  | Discussions and limitations: presents coherent and plausible arguments |
| In your opinion, are the above criteria sufficiently rigorous? | Yes = 1; No = 0 If not: article excluded |
