# Supplementary material for: How does nursing-sensitive indicator feedback with nursing or interprofessional teams work and shape nursing performance improvement systems? A rapid realist review
Source: Syst Rev. 2022 Aug 24;11:177. doi: 10.1186/s13643-022-02026-y (PMC9404638; doi:10.1186/s13643-022-02026-y)
Supplement: Supplementary file 4 — Additional file 4. Extraction form. [file 13643_2022_2026_MOESM4_ESM.docx]

**additional file 4**

*Extraction form*

| Main purpose of the article | a) Theoretical or conceptual model of nursing performance improvement systems |
| --- | --- |
|  | b) Operationalization or testing of performance measurement indicators or tools |
|  | c) Performance measurement framework or tool implementation analysis |
|  | d) Other; Specify: |
| Does the article present an explicit theoretical or conceptual model? | Yes = 1; No = 0 |
| Title of model |  |
| Graph of model | Yes = 1; No = 0 |
| Key dimensions of model (if no graph) |  |
| Who are the actors identified and what are their roles, interests and links (connections: strength or density)? |  |
| What knowledge do the actors have about feedback shared with teams? |  |
| What material objects and their attributes are identified in the transmission of performance results to the teams (e.g. dashboard, cockpit manager, report, resources)? |  |
| What symbolic objects and their attributes are identified in the transmission of performance results to the teams (e.g. values, standards, rules, knowledge)? |  |
| Are strategic positions highlighted (e.g. mediator, intermediary)? |  |
| Are power relationships highlighted? |  |
| Were new roles developed? Were new actors introduced into the system? |  |
| Are system reorganizations or adaptations highlighted? |  |
| Are points of convergence or controversy, issues or uncertainties highlighted? |  |
| Are changes following feedback highlighted? Are adherences to the system highlighted? |  |
| Are one or more translation operations identified?  Yes = 1; No = 0 | Problematization |
|  | Interessement |
|  | Enrolment |
|  | Mobilization |
| Problematization: Arguments relating to the advantages and disadvantages of transmitting the performance results to the teams |  |
| Problematization: Methods (e.g. negotiations and adjustments) and actions (e.g. mediators) |  |
| Interessement: Strategies applied to encourage actors to join the network and to strengthen alliances (e.g. training, resources, convergences) |  |
| Enrolment: Negotiation of roles, alignment of actors, shifts in position related to the transmission of performance results to the teams |  |
| Mobilization: Finalization (e.g. common goal, concessions, coordination of several actors), interiorization (e.g. meaning, belonging), transfer of responsibility (e.g. no longer project manager or proponent), formalization (e.g. rules, standards) |  |
| What variables can be used to test the theory? |  |
| What are the results of the test? |  |
| What are the limitations of the test? |  |
| Are relevant references and links to websites identified? If so, list them (reference number or site acronym). |  |
| Comments (e.g. the article’s contribution with respect to the project’s objectives) |  |
