# Supplementary material for: How does nursing-sensitive indicator feedback with nursing or interprofessional teams work and shape nursing performance improvement systems? A rapid realist review
Source: Syst Rev. 2022 Aug 24;11:177. doi: 10.1186/s13643-022-02026-y (PMC9404638; doi:10.1186/s13643-022-02026-y)
Supplement: Supplementary file 5 — Additional file 5. Greenhalgh et al.’s logic model. [file 13643_2022_2026_MOESM5_ESM.docx]

**additional file 5**

Data organization form inspired from Greenhalgh et al.’s original logic model

| Stage | Questions |
| --- | --- |
| *Audit* | How does the process of choosing indicators and targets work? How does the data management process work?  How does the data editing process work?  Who is involved?  In what context? |
| Data feedback | How does the data feedback process work?  Who is involved?  In what context |
| *Data contestation* | Are there any contesting data?  How does the process of contesting data work?  Who is involved?  In what context? |
| Interpret data | How does the process of interpreting data work?  Who is involved?  In what context? |
| Identify cause of problem | How does the process to identify cause of problem work?  Who is involved?  In what context |
| Develop a solution | How does the process to develop a solution work?  Who is involved?  In what context |
